# EEG microstate as a biomarker of post-stroke depression with acupuncture treatment

**DOI:** 10.3389/fneur.2024.1452243

**Published:** 2024-10-29

**Authors:** Conghui Wei, Qu Yang, Jinling Chen, Xiuqin Rao, Qingsong Li, Jun Luo

**Affiliations:** Department of Rehabilitation Medicine, The Second Affiliated Hospital of Nanchang University, Nanchang, China

**Keywords:** post-stroke depression, acupuncture, microstate, EEG, XGBoost model

## Abstract

**Background:**

Post-stroke depression (PSD) is a prevalent psychiatric complication among stroke survivors. The PSD researches focus on pathogenesis, new treatment methods and efficacy prediction. This study explored the electroencephalography (EEG) microstates in PSD and assessed their changes after acupuncture treatment, aiming to find the biological characteristics and the predictors of treatment efficacy of PSD.

**Methods:**

A 64-channel resting EEG data was collected from 70 PSD patients (PSD group) and 40 healthy controls (HC group) to explore the neuro-electrophysiological mechanism of PSD. The PSD patients received 6 weeks of acupuncture treatment. EEG data was collected from 60 PSD patients after acupuncture treatment (MA group) to verify whether acupuncture had a modulating effect on abnormal EEG microstates. Finally, the MA group was divided into two groups: the remission prediction group (RP group) and the non-remission prediction group (NRP group) according to the 24-Item Hamilton Depression Scale (HAMD-24) reduction rate. A prediction model for acupuncture treatment was established by baseline EEG microstates.

**Results:**

The duration of microstate D along with the occurrence and contribution of microstate C were reduced in PSD patients. Acupuncture treatment partially normalized abnormal EEG microstates in PSD patients. Baseline EEG microstates predicted the efficacy of acupuncture treatment with an area under the curve (AUC) of 0.964.

**Conclusion:**

This study provides a novel viewpoint on the neurophysiological mechanisms of PSD and emphasizes the potential of EEG microstates as a functional biomarker. Additionally, we anticipated the therapeutic outcomes of acupuncture by analyzing the baseline microstates, which holds significant practical implication for the PSD treatment.

## Introduction

PSD is an abnormal manifestation of mood depression, loneliness, and sleep disturbance in stroke patients (1, 2). Epidemiological studies indicate that approximately 31% of stroke patients experience varying degrees of depressive symptoms (3, 4). The mental status of PSD patients can significantly affect their physical recovery and life quality. Additionally, the severity of depression correlates with stroke severity and mortality rates (5). In clinical practice, depressive symptoms are commonly alleviated by taking monoamine oxidase inhibitors, 5-hydroxytryptamine (5-HT) reuptake inhibitors and tricyclic antidepressants (6). However, the complex pathogenesis and susceptible recurrence of PSD, combined with potential toxic side effects from prolonged medication use, often undermine patients’ adherence to treatment (7). Therefore, it is imperative to employ alternative methods to more effectively manage depressive symptoms in PSD patients.

Various viable alternatives have been investigated for the treatment of PSD, among which acupuncture has been widely adopted globally for its efficacy and safety (8). In China, acupuncture is recommended as a complementary therapy for both PSD and post-stroke insomnia (PSI) (9). Functional magnetic resonance imaging (fMRI) studies have demonstrated that acupuncture modulates the interconnected dysfunctions associated with depressive symptoms, thereby facilitating sustained clinical improvement (10).

Individual differences in resting-state brain functional connectivity are associated with different types of depression, which shown by previous fMRI examinations (11, 12). Recent research indicates that EEG microstates can distinguish between depressive subtypes (13). EEG microstates can reflect dynamic changes in large-scale brain networks, characterized as semi-stable, transient voltage topographies that recur during resting-state EEG recordings (14). Persistent repetitive microstates likely arise from repeated co-activation of interconnected brain regions, each lasting approximately 80 ms before transitioning to another temporarily stable pattern. The four typical microstates (A-D) were present in almost all subjects, representing the synchronized activity within large-scale network nodes (15).

On the relationship between EEG microstates and the treatment of depression, Damborsk et al. (16) demonstrated a significant positive correlation between the microstates occurrence and medication amount. The usage of antipsychotics, antidepressants, and mood stabilizers was markedly associated with the occurrence of microstate E. Additionally, another study indicated that variations in the duration of microstate C and microstate D were strongly predictors of the response to electroconvulsive therapy (ECT) (17). Therefore, EEG microstates hold promise as biomarkers for assessing the effectiveness of therapeutic interventions in various neurological disorders.

Previous studies have primarily examined the differences between PSD patients and normal subjects, with limited focus on the prediction of depression efficacy (18, 19). Therefore, we investigated the EEG microstates of PSD patients and evaluated the changes of EEG microstates after acupuncture treatment to identify the biological characteristics and the predictors of PSD treatment efficacy.

## Methods

### Participants

**Figure 1:**
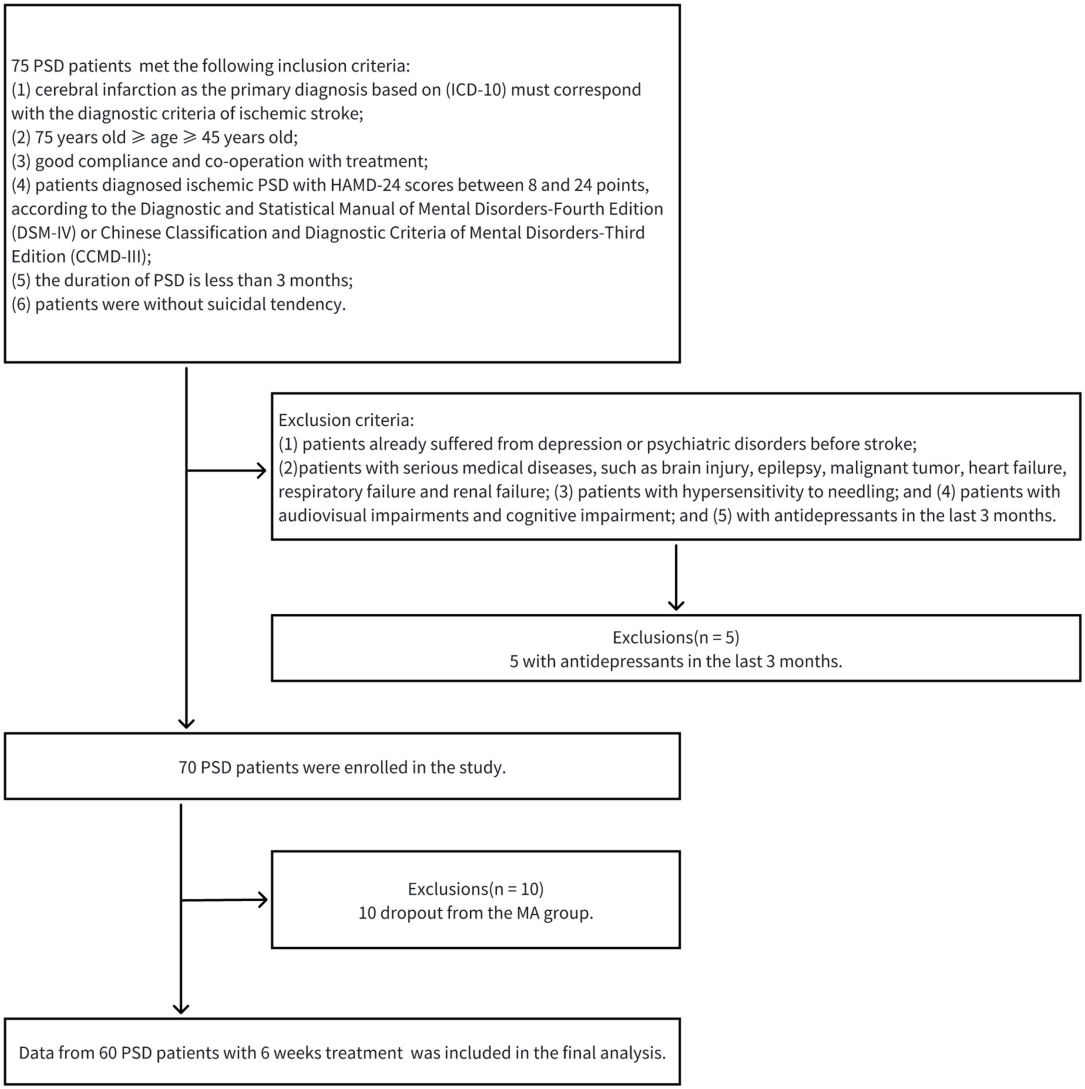
A schematic representation of patients’ therapy processes.

Between October 2022 and May 2023, this single-center clinical trial treated and assessed PSD patients for 6 weeks at the Department of Rehabilitation Medicine of the Second Affiliated Hospital of Nanchang University. This study underwent a review and approval process by the institutional ethics committee of the Second Affiliated Hospital of Nanchang University (BR/AF/SG-04/1.0) before subject enrolment. The research was registered with the Chinese Clinical Trial Registry (ChiCTR2200065112). Before the clinical trial, every participant provided their signature on an informed consent document. 75 PSD patients met the inclusion criteria and 5 PSD patients with antidepressants in the last 3 months were excluded. In the 70 PSD patients, 60 completed the 6-week course of acupuncture treatment who did not receive any treatment at baseline. PSD patients with acupuncture treatment for 6 weeks were designated as the MA group. EEG data was obtained from PSD group (*n* = 70), MA group (*n* = 60) and HC group (*n* = 40). Figure 1 shows a schematic representation of the patients’ therapy processes.

The inclusion criteria are as follows: (1) cerebral infarction as the primary diagnosis based on (ICD-10) must correspond with the diagnostic criteria of ischemic stroke (20); (2) 75 years old ≥ age ≥ 45 years old; (3) good compliance and co-operation with treatment; (4) patients diagnosed ischemic PSD with HAMD-24 scores between 8 and 24 points, according to the Diagnostic and Statistical Manual of Mental Disorders-Fourth Edition (DSM-IV) (21) or Chinese Classification and Diagnostic Criteria of Mental Disorders-Third Edition (CCMD-III) (22); (5) the duration of PSD is less than 3 months; and (6) patients were without suicidal tendency. The exclusion criteria are as follows: (1) patients with depression or psychiatric disorders history before stroke; (2) patients with serious medical diseases, such as brain injury, epilepsy, heart failure, renal failure and malignant tumor; (3) patients with hypersensitivity to needling; and (4) patients with audiovisual impairments and cognitive impairment; and (5) with antidepressants in the last 3 months. The termination criteria are as follows: (1) patients with poor compliance; (2) patients who voluntarily withdrew; and (3) patients with serious adverse reactions or stroke recurrence.

### Treatment

PSD patients received 30 sessions of conventional rehabilitation over 6 consecutive weeks (5 times per week for 30 min). Acupuncture was performed with sterile disposable needles (0.30 mm × 40 mm). MA was conducted based on a standard brain-opening stabbing protocol with a fixed angle, depth, frequency, and retention time. Patients in the MA group received acupuncture at 5 fixed acupoints: the Baihui acupoint, the Shenting acupoint, the Yintang acupoint, the Temple acupoint and the Four Divine acupoint. After needle insertion, the needles were retained for 30 min.

PSD patients also receive necessary symptomatic treatment for primary disease (including blood pressure control, glucose reduction, lipid lowering, anticoagulant therapy, antiplatelet therapy and intracranial decompression), are offered nutritional support, and are provided with prevention and treatment of complications (respiratory and urinary tract infections, etc.).

### Clinical assessment

The depression degree was evaluated using the HAMD-24 scale, which comprises 24 questions, including mental anxiety, depressed mood, guilt, suicide, work and interest, sleep, and self-awareness. This scale is intended for adults with depressive symptoms. It can be used to assess depressive symptoms in a variety of disorders such as depression, bipolar disorder, and neurological disorders (23). The severity of depressive symptoms was assessed for each item using a scale that ranged from 0 to 4, with higher scores correspond to more severe symptoms. In our study, treatment effects were also assessed based on the HAMD-24 scores. No therapeutic effect was defined as the patients’ symptomatic manifestations not improving or even aggravating after treatment, and the HAMD-24 score was reduced by <30% compared with that before treatment. A significant therapeutic effect was defined as the patients’ clinical symptoms basically disappeared after treatment, and the HAMD-24 score was reduced by >30% or HAMD-24 score < 7. Based on the above criteria, the MA group was divided into the RP and NRP groups.

### EEG recording and pre-processing

In an electrically shielded dark room, subjects were instructed to remain calm and sit upright in a comfortable position. EEG data was collected for 5 min with subjects awake and eyes open. All subjects were monitored during the experiment and recording was stopped if there were signs of drowsiness. A 64-channel system (Nuroscan EEG System, Australia) with a 1 kHz sampling rate was used to record EEG data. M1 and M2 electrodes in mastoid were used as reference electrodes during acquisition, with 64 electrodes placed according to the international 10–20 system and the impedance of scalp electrodes was kept below 5 kΩ before recording.

EEG data underwent band-pass filtering with a frequency range of 1–30 Hz (24). Infomax-based independent component analysis (ICA) was then used to remove artefacts from ECG and eye movements, and one or two channels were excluded due to excessive artefacts (25). Based on the waveform topography and time course, only components associated with eye movements, blinks, and ECG were removed. After artefact cleaning, EEG data was interpolated using 3D spherical spline for previously identified noisy channels and re-referenced using a whole-brain averaged reference. Every preprocessing step was performed utilizing the EEGLAB toolbox of MATLAB (V.R2019a, United States) (26).

### Microstate analysis

In this study, microstate analysis was performed utilizing the EEGLAB toolbox of MATLAB (V.R2019a, United States) (26). The data were analyzed using the atomize and agglomerate hierarchical clustering (AAHC) algorithm and referenced to the four classical microstates proposed by Koenig (27, 28). Topographic map polarity was ignored in the clustering process. For the purpose of improving the ratio between the signal and the noise, only time-point data with local maxima of global field power (GFP) were clustered (29). Since the EEG topography remains stable near the peak of GFP, and the topography changes at trough values, the peak point is the time point with the highest signal-to-noise ratio. To avoid repeated selection of the same GFP peak, the minimum time distance between two GFP peaks was set to 10 ms. 1,000 GFP peaks were randomly selected from the EEG data of each subject to extract EEG topographic clustering samples. GFP, an indicator of the scalp potential field’s strength, was calculated using the following formula:


$$GFP=\sqrt{\frac{\sum_{i=1}^{n}u_{i}^{2}}{n}}$$

*n* represents the number of electrodes, u represents the voltage value, and *i* represents the *i*-th electrode.

Cluster analyses were initially calculated at the individual level and subsequently at the group level. The AAHC algorithm first defines the brain topography at each GFP peak as a template mapping of the cluster. Then, the “worst” clusters are selected, by which we mean that the culling of the cluster is done at the lowest “cost”. This selection is done by identifying the cluster with the smallest global explained variance (GEV). GEV was calculated by summing the explanatory variances of each microstate, weighted by GFP, to evaluate the extend to which representative microstate topographies describe the raw EEG data. A higher value of GEV indicates a higher degree of interpretability of the EEG microstate template for the EEG activity of the original EEG signal.GEV is defined as:


$${GEV}_{n}=\frac{{\left(\mathrm{Corr}\left(x_{n},a_{n}\right)\times {GEV}_{n}\right)}^{2}}{\sum_{n}^{N},{{GEV}_{n}}^{2}}$$

x_n_ is the nth EEG time sampling point and a_n_ is the microstate prototype topography for the nth EEG microstate class. Corr is the correlation computed between the two EEG topographies.

The “worst” cluster is then atomised, which means that the brain topographies of this cluster are “freed” and no longer belong to any cluster. If there are multiple topographies in this “free” cluster, these “free” topographies are reassigned one at a time by calculating the spatial correlation between each “free” brain topography and the center of mass of each surviving class. Topographies were reassigned to the cluster with the highest spatial correlation. In addition, this cluster is considered to be the cluster with the highest correlation coefficient. The method proceeds recursively by removing classes one at a time and stops when only a single final class remains (even if the latter is useless).

The parameters were often statistically analyzed, including duration, occurrence, contribution, and transition probability, which reflected the information of neural activities:

1. Duration: It was defined as the mean length of the microstate. For the calculation, all the identical and adjacent initial prototype maps were assigned to one sequence. Then the sequence was calculated from the first initial prototype map to the last one;
2. Occurrence: It was defined as the number of occurrences of each microstate per second. This may reflect a tendency for potential neurons or nervous systems to be activated in that microstate;
3. Contribution: It was defined as the time of a microstate as a relative percentage of the total time;
4. Transition probability: It was defined as the likelihood of a microstate transforming randomly to other states. OrgTM_X > Y denotes the probability of switching from X to Y.

### Building predictive model

Pre-treatment EEG microstate characteristics (duration, occurrence, contribution and transition probability) of 40 PSD participants in the RP group and 20 in the NRP group were used as features to construct dataset. Before constructing the prediction model, the average contribution of each feature was calculated using the Extreme Gradient Boosting (XGBoost) model, and the top 10 important features were listed. XGBoost is a Boosting integrated tree model, and the core idea is to integrate multiple weak classifiers into one strong classifier. And it has the advantages of high prediction accuracy, fast training speed, high flexibility, and support for custom loss functions. Therefore, XGBoost is increasingly used in a wide range of clinical applications, including disease prognosis, efficacy prediction, and risk prediction (30, 31). In our study, the XGBoost model was configured with a learning rate of 0.01 and a maximum depth of 6. The data was subsequently partitioned into five equal sections, with one of them being selected as the test set. The remaining four parts were employed as the training set and were repeated five times. F1 score, AUC, specificity, accuracy and sensitivity were employed to assess model performance. AUC, reflecting overall predictive performance, approaches 1 with better performance. The items of XGBoost model were seen in Table 1. The model building process was conducted using R version 3.6.3 and Python version 3.7. Extreme Smart Analysis platform provided support for this work.^1^

**Table 1:** The XGBoost model building.

Items	Details
Features	Duration (A–D), Occurrence (A–D), Contribution (A–D),OrgTM_A > B, OrgTM_A > B, OrgTM_A > C, OrgTM_A > D, OrgTM_B > A, OrgTM_B > C, OrgTM_B > D, OrgTM_C > A, OrgTM_C > B, OrgTM_C > D, OrgTM_D > A, OrgTM_D > B, OrgTM_D > C
Parameters	learning_rate: 0.01, max_depth: 6, min_child_weight: 2, reg_lambda: 1
Verification method	5 fold cross-validation
Test set ratio	0.2
Evaluations	F1 score, AUC, specificity, accuracy, and sensitivity

### Statistical analyses

IBM SPSS Statistics version 24.0 (IBM Corp., Armonk, NY, United States) was employed to conduct statistical analyses. The age, education, PSD duration, and HAMD-24 scores were compared using the independent sample *t*-test, while the sex was compared using the chi-square test. The parameters (duration, occurrence, contribution, and transition probability) of each microstate were subjected to independent sample *t*-tests and correction were implemented utilizing the false discovery rate (FDR). Correlation analysis of microstate parameters with HAMD-24 scores in PSD group was performed using Pearson correlation. The test level was set at *α* = 0.05.

## Results

### Demographic information

Demographic information of the HC and PSD groups was presented in Table 2. Significant differences in age, sex, education were not observed between the two groups. The demographic information between the HC and PSD groups was comparable.

**Table 2:** Demographic information of the HC and PSD groups.

Characteristic	HC (*n* = 40)	PSD (*n* = 70)	t/χ^2^	*p*
Age (year)	58.90 ± 4.91	56.93 ± 6.65	1.64	0.11
Sex			0.16	0.69
Male	21 (52.50)	34 (48.57)		
Female	19 (47.50)	36 (51.43)		
Education (year)	7.10 ± 2.31	7.16 ± 2.57	−0.12	0.91

Demographic information of the RP and NRP groups was presented in Table 3. Significant differences in age and sex were not observed between the two groups. Significant differences in the education (*t* = 2.04, *p* = 0.046) and PSD duration (*t* = −3.15, *p* = 0.003) were found between the RP and NRP groups.

**Table 3:** Demographic information of the RP and NRP groups.

Characteristic	RP (*n* = 40)	NRP (*n* = 20)	t/χ^2^	*p*
Age (year)	58.03 ± 6.36	58.15 ± 6.46	−0.07	0.94
Sex			1.21	0.27
Male	20 (50)	7 (35)		
Female	20 (50)	13 (65)		
Education (year)	7.65 ± 2.72	6.20 ± 2.33	2.04	0.046*
PSD duration (day)	69.25 ± 4.20	73.15 ± 5.10	−3.15	0.003**

* *p* < 0.05; ** *p* < 0.01.

### HAMD-24 scores between the HC, PSD and MA groups

**Figure 2:**
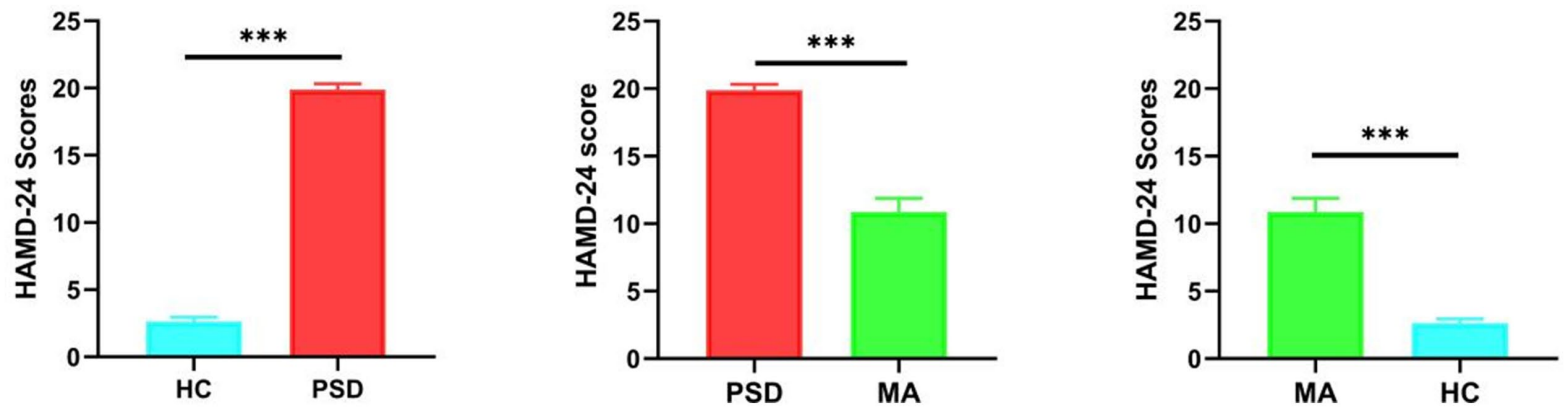
HAMD-24 scores between the HC (blue, *n* = 40), PSD (red, *n* = 70) and MA (green, *n* = 60) groups. The HC group means ± standard deviations is 2.63 ± 1.03. The PSD group means ± standard deviations is 19.89 ± 1.81. The HC group means ± standard deviations is 10.87 ± 3.93. *** *p <* 0.001.

Significant difference in the HAMD-24 scores was found between the HC and PSD groups (*t* = −55.23, *p* < 0.001). The mean change in HAMD-24 score from PSD group (19.89 ± 1.81) to MA group (10.87 ± 3.93) was −9.02 points (*t* = 17.19, *p* < 0.001). Following acupuncture treatment, PSD patients showed noticeable improvement, as indicated by a reduction in HAMD-24 scores (Figure 2). Meanwhile, there was also a significant difference between HC group (2.63 ± 1.03) and MA group (*t* = −12.95, *p* < 0.001). These results suggested that acupuncture significantly improved depressive symptoms in PSD patients.

### Four similar sets of microstates

**Figure 3:**
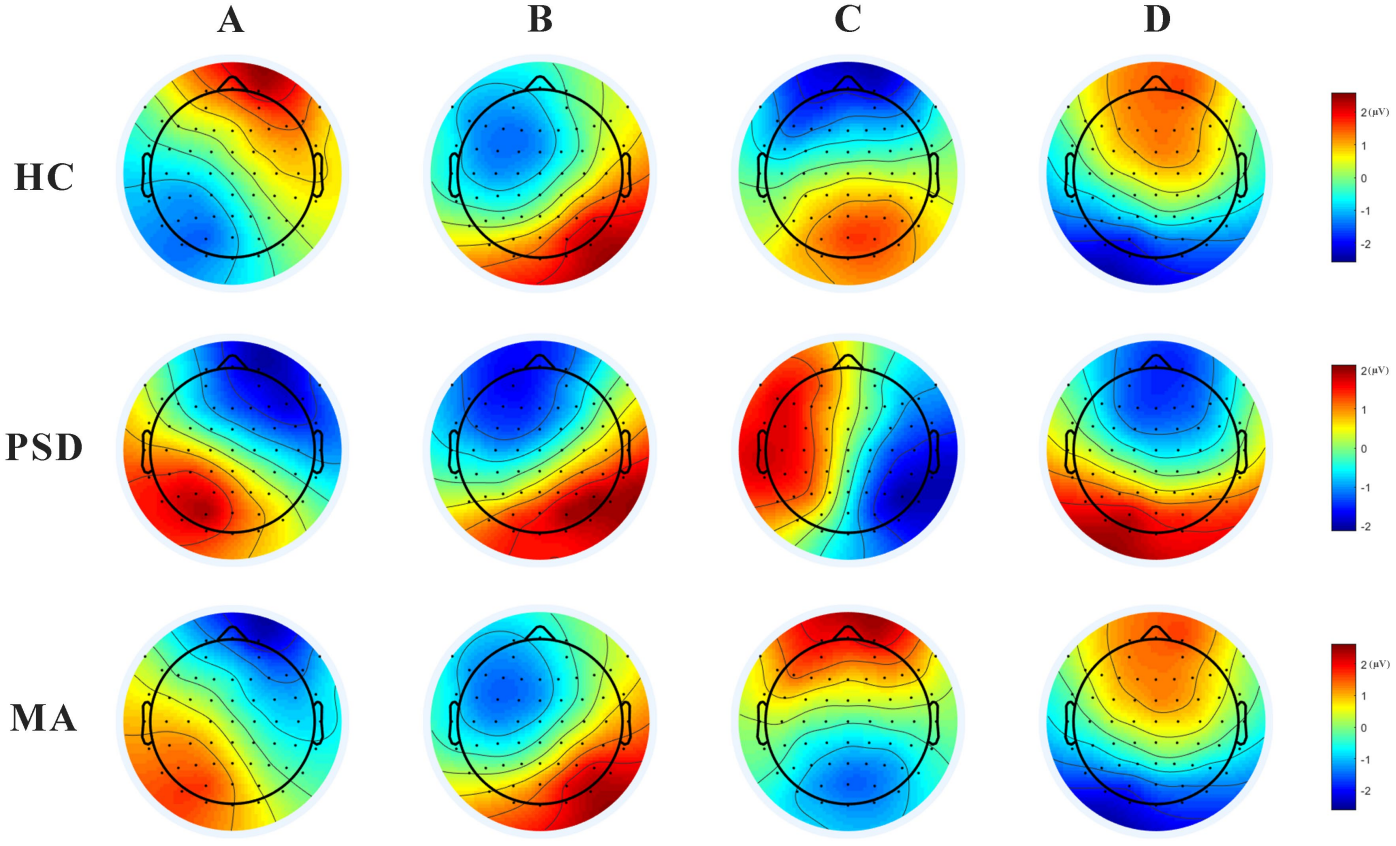
The 4 scalp maps in the HC (*n* = 40), PSD (*n* = 70), and MA groups (*n* = 60). Scalp topographies revealed left posterior-right anterior orientation (microstate A), right posterior-left anterior orientation (microstate B), anterior–posterior orientation (microstate C), and fronto-central maximum (microstate D).

Microstate analysis showed four microstates across HC, PSD, and MA groups (Figure 3). Based on previous reports (28, 32), the four scalp maps were assigned labels A to D. Scalp topographies revealed left posterior-right anterior orientation (microstate A), right posterior-left anterior orientation (microstate B), anterior–posterior orientation (microstate C), and fronto-central maximum (microstate D). The variance ratios for these four types of topographic map interpretations ranged from 70 to 84%.

### Differences in microstates between HC and PSD groups

**Figure 4:**
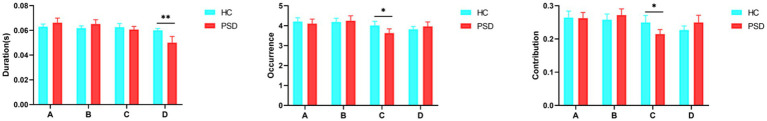
The parameters of microstates in the HC (blue, *n* = 40) and PSD (red, *n* = 70) groups. The parameters in the figure from left to right are duration, occurrence, and contribution in that order. * *p <* 0.05. ** *p <* 0.01.

Concerning microstates duration, microstate D was shorter in the PSD group than in the HC group (*t* = 2.99, *p* = 0.003). However, no significant difference was found for microstates A, B, and C (Figure 4). The occurrence (*t* = 2.39, *p* = 0.02) and contribution (*t* = 2.98, *p* = 0.004) of microstate C were both significantly lower in the PSD group than in the HC group. Regarding transition probabilities of microstates, significant differences were observed in OrgTM_A > C, OrgTM_C > A, OrgTM_B > C, OrgTM_C > B, OrgTM_D > B (Table 4).

**Table 4:** Transition probabilities of microstates in the HC and PSD groups.

Transition types	*p*	Transition types	*p*
OrgTM_A > B	0.20	OrgTM_C > A	<0.001***
OrgTM_A > C	<0.001***	OrgTM_C > B	<0.001***
OrgTM_A > D	0.34	OrgTM_C > D	0.53
OrgTM_B > A	0.13	OrgTM_D > A	0.10
OrgTM_B > C	0.003**	OrgTM_D > B	0.02*
OrgTM_B > D	0.071	OrgTM_D > C	0.49

* *p* < 0.05; ** *p* < 0.01; *** *p* < 0.001.

### Acupuncture treatment reversed the partial abnormal microstate in PSD patients

**Figure 5:**
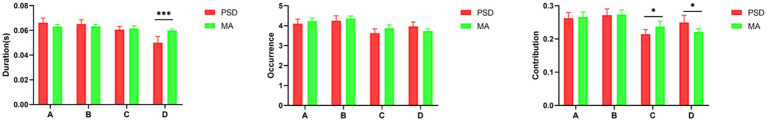
The parameters of microstates in the PSD (red, *n* = 70) and MA (green, *n* = 70) groups. The parameters in the figure from left to right are duration, occurrence, and contribution in that order. * *p <* 0.05. *** *p <* 0.001.

The parameters of microstates in the PSD and MA groups were shown in Figure 5. After acupuncture treatment, the duration of microstate D was significantly longer in the MA group compared to the PSD group (*t* = − 3.60, *p* = 0.0001). The contribution of microstate C was higher in the MA group compared with the PSD group (*t* = − 2.13, *p* = 0.03). However, the contribution of microstate D was higher in the PSD group than in the MA group (*t* = 2.31, *p* = 0.02). Regarding transition probabilities of microstates, significant differences were found in OrgTM_A > C, OrgTM_C > A, OrgTM_B > C, OrgTM_C > B, OrgTM_D > B (Table 5). These findings indicated that acupuncture significantly reversed the partial abnormal microstate in PSD patients.

**Table 5:** Transition probabilities of microstates in the PSD and MA groups.

Transition types	*p*	Transition types	*p*
OrgTM_A > B	0.87	OrgTM_C > A	0.003**
OrgTM_A > C	0.002**	OrgTM_C > B	<0.001**
OrgTM_A > D	0.07	OrgTM_C > D	0.10
OrgTM_B > A	0.56	OrgTM_D > A	0.60
OrgTM_B > C	<0.001**	OrgTM_D > B	0.04*
OrgTM_B > D	0.28	OrgTM_D > C	0.11

* *p* < 0.05; ** *p* < 0.01; *** *p* < 0.001.

### Correlations of microstate parameters and HAMD-24 scores in PSD group

Correlation analysis between microstate parameters and HAMD-24 scores in PSD group (*n* = 70) was performed. The contribution of microstate C (*r* = −0.28, *p* = 0.028) and OrgTM_B > C (*r* = −0.28, *p* = 0.028) were negatively correlated with HAMD-24 scores. Contrarily, OrgTM_A > C (*r* = 0.35, *p* = 0.003) and OrgTM_C > A (*r* = 0.38, *p* < 0.001) were positively correlated with HAMD-24 scores. Other microstate parameter were not significantly correlated (Table 6).

**Table 6:** Correlations of microstate parameters and HAMD-24 scores in PSD group.

Microstate parameter	*r*	*p*	Microstate parameter	*r*	*p*
Duration_A	0.19	0.11	OrgTM_A > B	0.059	0.62
Duration_B	0.17	0.17	OrgTM_A > C	0.35	0.003**
Duration_C	0.13	0.28	OrgTM_A > D	−0.088	0.47
Duration_D	0.17	0.16	OrgTM_B > A	0.062	0.61
Occurrence_A	−0.12	0.31	OrgTM_B > C	−0.24	0.047*
Occurrence_B	−0.12	0.30	OrgTM_B > D	−0.14	0.26
Occurrence_C	−0.22	0.07	OrgTM_C > A	0.38	<0.001***
Occurrence_D	−0.23	0.055	OrgTM_C > B	−0.089	0.46
Contribution_A	0.062	0.61	OrgTM_C > D	0.013	0.91
Contribution_B	0.003	0.98	OrgTM_D > A	−0.12	0.32
Contribution_C	−0.28	0.02*	OrgTM_D > B	−0.05	0.68
Contribution_D	0.001	0.99	OrgTM_D > C	−0.003	0.98

* *p* < 0.05; ** *p* < 0.01; *** *p* < 0.001.

### Prediction of treatment outcome from baseline EEG microstates

**Figure 6:**
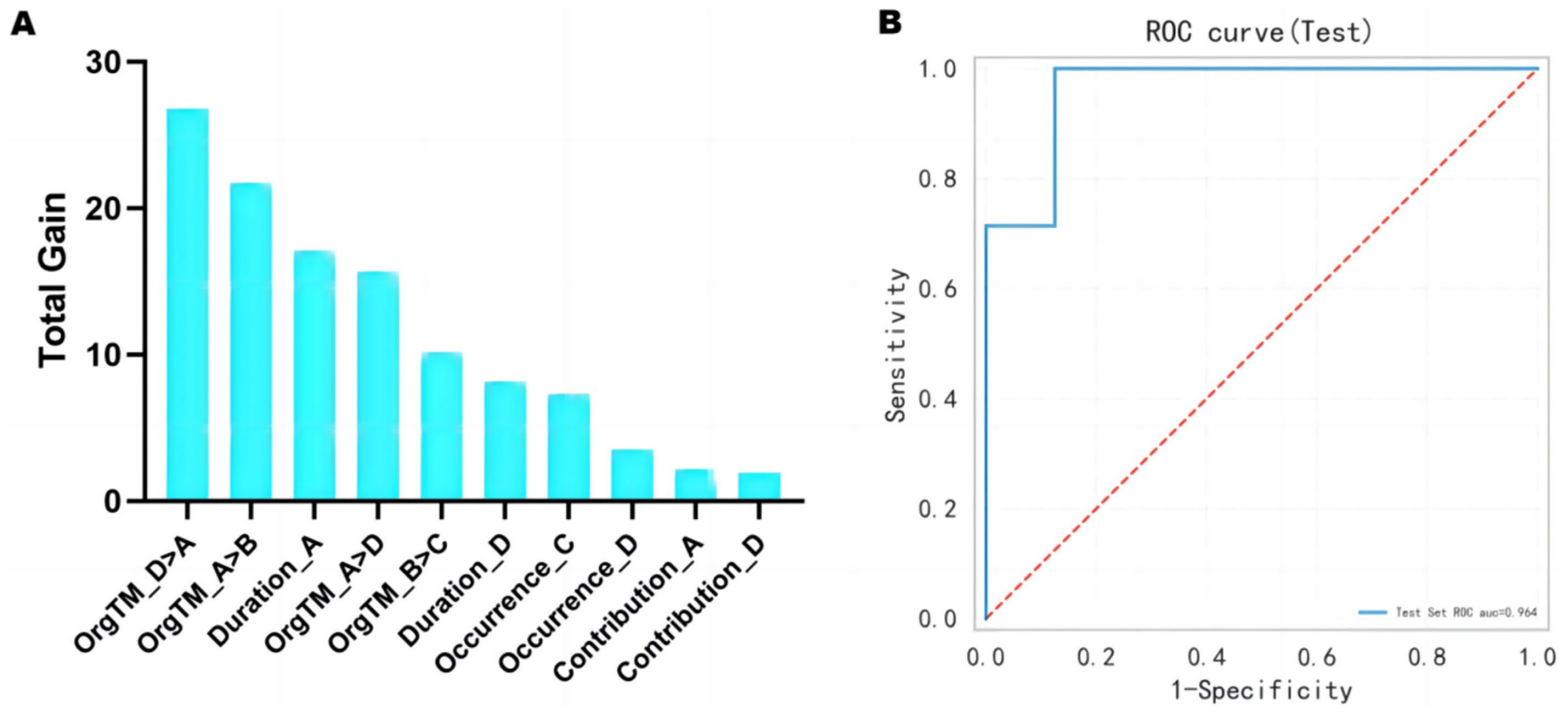
Treatment outcome prediction from baseline EEG microstates. **(A)** The top 10 most important features of the model. **(B)** The ROC curve of the test set is 0.964.

Figure 6A displays the top 10 most important features of the prediction model. The 10 features with the highest total gain (in descending order) are: OrgTM_D > A, OrgTM_A > B, Duration_A, OrgTM_A > D, OrgTM_B > C, Duration_D, Occurrence_C, Occurrence_D, Contribution_A, Contribution_D. The ROC curve of the test set displays in Figure 6B. The prediction performance of the XGBoost model are as follows: AUC = 0.964, accuracy = 0.80, sensitivity = 0.714, specifcity = 0.875, and F1 score = 0.769.

## Discussion

The results of this study carry several significant implications. First, the PSD group exhibited a notable reduction in the duration of microstate D along with the occurrence and contribution of microstate C when compared with the HC group. Second, acupuncture treatment enhanced the duration of microstate D and partially normalized microstate C in PSD patients. Finally, this study indicated that the baseline characteristics of microstates in PSD patients could predict the alleviation of depressive symptoms following acupuncture therapy.

Spatial patterns that corresponded to well-known resting-state networks were detected in the four canonical microstate classes. Additionally, the activation of the auditory, visual, salience and attention networks was associated with the intra-individual fluctuations of microstates A, B, C and D, respectively (33). Various neurological and mental disorders, including schizophrenia and Lewy body dementia, have been associated with abnormal EEG microstates, which may suggest a disturbance in the brain’s complex dynamic properties (34, 35). This disruption may result in a reduction in the adaptability and flexibility that are essential for normal function of the brain (36). Our study revealed that PSD patients experienced a shorter duration of microstate D than the HC group, suggesting that clinical depression can impair both cognitive control and attention (37, 38). Notably, a previous study found that patients with PSD showed a higher frequency of microstate D and a shorter average length of microstate C (39). Nevertheless, these findings need further validation, taking into account variations in the number of clusters and the timing of resting-state EEG acquisition.

Studies using source imaging techniques or combined EEG-fMRI have reported that the active regions of microstate C partially overlap with the DMN (Default Mode Network), the “self-experience” sub-network (40, 41) and the salience network (33). In one study (42), repetitive transcranial magnetic stimulation was applied to brain regions belonging to the DMN and the dorsal attentional network. After stimulation of the angular gyrus and the intraparietal sulcus, the topology of microstate C changed significantly compared to the pre-stimulation period, which was not seen with sham stimulation or stimulation of the temporoparietal junction. Kikuchi et al. (43) quantified EEG microstates in schizophrenia patients before and after treatment with antipsychotic medication. They reported a significant positive correlation between symptomatic changes and the occurrence of microstate C. Therefore, EEG microstates hold promise as biomarkers for assessing the effectiveness of therapeutic interventions in various neurological disorders.

Source localisation and combined EEG-fMRI studies have reported consistent results, mainly in areas overlapping with the fronto-parietal network (33, 40, 41). Furthermore, one study reported an increase in temporal features after repetitive transcranial magnetic stimulation of the intraparietal sulcus, a key hub of the dorsal attention network (42). Thus, microstate D is primarily associated with executive processes such as working memory, cognitive control, attentional redirection and detection of behaviourally relevant stimuli. Multiple studies have reported an increase in the presence of microstate D during arithmetic tasks, where participants are required to count backwards from a seed number, compared to task-free rest and/or autobiographical memory tasks (40, 44). This is further supported by the increased presence of microstate D during a recognition task (45), during subsequent rest and sleep after virtual maze training (46), and during a spatial relations task (47). The dysfunctional brain activity is a significant factor in the development of depression, which can potentially interfere with the shifts in EEG microstates. EEG microstates can be influenced by changes in the overall coordinated pattern of functional brain activity. Our research presented empirical support that the occurrence and contribution of aberrant microstate C and microstate D were enhanced by the treatment of PSD patients with acupuncture.

Significant differences in age and sex were not observed between the RP and NRP groups. Significant differences in the education and PSD duration were found between the RP and NRP groups. The significant effects of sex and age on the effectiveness of acupuncture treatment have not been clearly indicated in previous studies (48, 49). One study suggests there may be a time-related therapeutic window for antidepressants, and early antidepressant treatment promotes daily mobility (50). Therefore, early antidepressant treatment provides better therapeutic results. Apart from patient factors and therapeutic window, issues such as needle insertion, needling sensation, psychological factors, acupoint specificity, acupuncture manipulation, and needle duration also have relevant influences on the therapeutic effects of acupuncture (51). A systematic review revealed that there have been relatively few research studies assessing the relationship between expectancy and treatment responses following acupuncture, and suggested future studies should assess the factors of efficacy (52).

Although acupuncture treatment can effectively alleviate depression symptoms in PSD patients, its effectiveness varies significantly among individuals. Hence, it is essential to differentiate between individuals who respond well to acupuncture (high-responders) and those who do not (low-responders) in order to optimize future treatment approaches. We employed a machine learning technique to estimate the effectiveness of therapy and observed that baseline microstate indicators in PSD patients could accurately forecast whether the depression symptoms could demonstrate significant improvement after a 6-week course of acupuncture. For low-responders, modifying the treatment modality or combining acupuncture with other therapies (such as cognitive-behavioral therapy or medication) could enhance therapeutic effectiveness (53). The top 10 most important features highlighted the crucial role of transitions between distinct microstates in the prediction model. This emphasizes the need to include other microstate indicators, such as OrgTM_D- > A, OrgTM_A- > B in future research.

There are several limitations in our study. First, the absence of follow-up assessments for PSD patients hindered the evaluation of the long-term effects of acupuncture treatment on PSD. Second, variations in microstate analysis methods could yield different outcomes. The clustering algorithms, examined bands, the number of maps used for backfitting, and whether all data points or only local maxima of the global field power were analyzed were all considered methodological differences (16). In this study, we utilized resting-state EEG rather than task-related EEG. Depression impacts not only mood and cognition but also motivational processes (54). Consequently, differences in task performance between healthy controls and patients may be influenced by various levels of motivation (55). The use of the resting state EEG has the potential to avoid task-related confounds.

Divergent findings could potentially be a reflection of the pathophysiological heterogeneity of depression. The experimental group in Strik’s study consisted of depressed patients with unipolar or bipolar mood disorders, which is similar to our current sample (56). A recent study was even limited to treatment-resistant depression. To advance understanding of depression’s microstate characteristics, future studies should expand their scope to encompass patients with diverse conditions such as bipolar depression and postpartum depression (57). In further research, our objective is to overcome these limitations and conduct further investigation on the pathogenesis of depression and the mechanisms by which acupuncture treatment works. This will provide additional perspectives and enhance approaches for treating depression.

## Conclusion

This study provides a novel viewpoint on the neurophysiological mechanisms of PSD and emphasizes the potential of EEG microstates as functional biomarkers. Additionally, we anticipated the therapeutic outcomes of acupuncture by analyzing the baseline microstates, which holds significant practical implications for PSD treatment.

## Data Availability

The raw data supporting the conclusions of this article will be made available by the authors, without undue reservation.

## Footnotes

^1^ https://www.xsmartanalysis.com/
